# Effects of metronome walking on long-term attractor divergence and correlation structure of gait: a validation study in older people

**DOI:** 10.1038/s41598-024-65662-5

**Published:** 2024-07-09

**Authors:** Sophia Piergiovanni, Philippe Terrier

**Affiliations:** Haute-Ecole Arc Santé, HES-SO University of Applied Sciences and Arts Western Switzerland, Espace de l’Europe 11, 2000 Neuchâtel, Switzerland

**Keywords:** Gait analysis, Aging, Nonlinear dynamics, Metronome walking, Biomedical engineering, Geriatrics, Ageing, Risk factors

## Abstract

This study investigates the effects of metronome walking on gait dynamics in older adults, focusing on long-range correlation structures and long-range attractor divergence (assessed by maximum Lyapunov exponents). Sixty older adults participated in indoor walking tests with and without metronome cues. Gait parameters were recorded using two triaxial accelerometers attached to the lumbar region and to the foot. We analyzed logarithmic divergence of lumbar acceleration using Rosenstein’s algorithm and scaling exponents for stride intervals from foot accelerometers using detrended fluctuation analysis (DFA). Results indicated a concomitant reduction in long-term divergence exponents and scaling exponents during metronome walking, while short-term divergence remained largely unchanged. Furthermore, long-term divergence exponents and scaling exponents were significantly correlated. Reliability analysis revealed moderate intrasession consistency for long-term divergence exponents, but poor reliability for scaling exponents. Our results suggest that long-term divergence exponents could effectively replace scaling exponents for unsupervised gait quality assessment in older adults. This approach may improve the assessment of attentional involvement in gait control and enhance fall risk assessment.

## Introduction

Falls in the older population are a major public health concern due to their high prevalence and associated adverse outcomes, including injury, loss of independence, and reduced quality of life^1,2^. The Swiss Council for Accident Prevention reports that falls occurring at home are responsible for approximately 1500 deaths in Switzerland each year among individuals over the age of 65^3^. Early identification of individuals at risk for falls is crucial. Effective strategies to reduce the incidence and adverse consequences of falls can be implemented once high-risk individuals are identified^4,5^. Paying special attention to identifying specific gait characteristics associated with an increased likelihood of stumbling and falling is essential, given that many falls occur during walking^6^.

Aging is associated with a decline in walking ability due to various physiological changes, such as weakened muscles and impaired motor control, resulting in a degraded gait quality^7^. While very old frail seniors often exhibit obvious signs of poor balance during ambulation, evaluating fall risk in healthy, active older persons is more complex. In clinical settings, fall risk assessment has traditionally relied primarily on questionnaires^8^ and one-time functional testing^9^. However, relying solely on short, subjective, and infrequent evaluations may not provide a comprehensive understanding of an individual’s daily fall risk. It is becoming increasingly evident that continuous monitoring of movements during daily activities can significantly enhance fall risk assessment^10,11^.

Over the past decade, there has been a surge of research focused on the use of accelerometers to identify older people at risk of falling, particularly in unsupervised assessments^12^. Technological advances in wearable inertial sensors now allow for high-frequency recording of body motion over extended periods of time. These advances facilitate the detection of walking bouts and analysis of gait patterns, shedding light on gait quality in everyday life^13–18^.

To monitor long-term mobility using a single inertial sensor, typically attached to the lower back, specific analysis methods are required. It is still necessary to conduct further research on the best way to assess fall risk in this context^19,20^. In fact, calculating standard gait parameters using a trunk-mounted accelerometer can be challenging. While average stride duration and stride length can be measured accurately^21^, it is often difficult outside the laboratory to detect gait events (e.g., toe-off events) in each step^22,23^.

As an alternative method not dependent on the detection of steps, the local dynamic stability (LDS) method^24,25^ has become increasingly popular in recent years for assessing aging gait and dynamic balance^20,26^. In the field of nonlinear dynamics, a branch of mathematics concerned with complex systems, researchers have developed the concept of Lyapunov exponents^27^. For a basic understanding, these exponents can be seen as quantifying the sensitivity of a system to small initial changes, reflecting LDS. Gait analysis has adopted this concept^24^ to investigate how tiny fluctuations during walking, such as those arising from muscle noise or uneven terrain, can influence a person’s overall gait stability. LDS method applies to continuous gait signals and is often assessed using trunk acceleration. LDS has been shown to be reliable under free-living conditions^28,29^ and has been successfully associated with fall risk in older people^13,30–32^. It is also a valuable tool for assessing gait quality in patients with various conditions such as Parkinson’s disease^33,34^, multiple sclerosis^34–37^, and stroke^34,38,39^.

**Figure 1:**
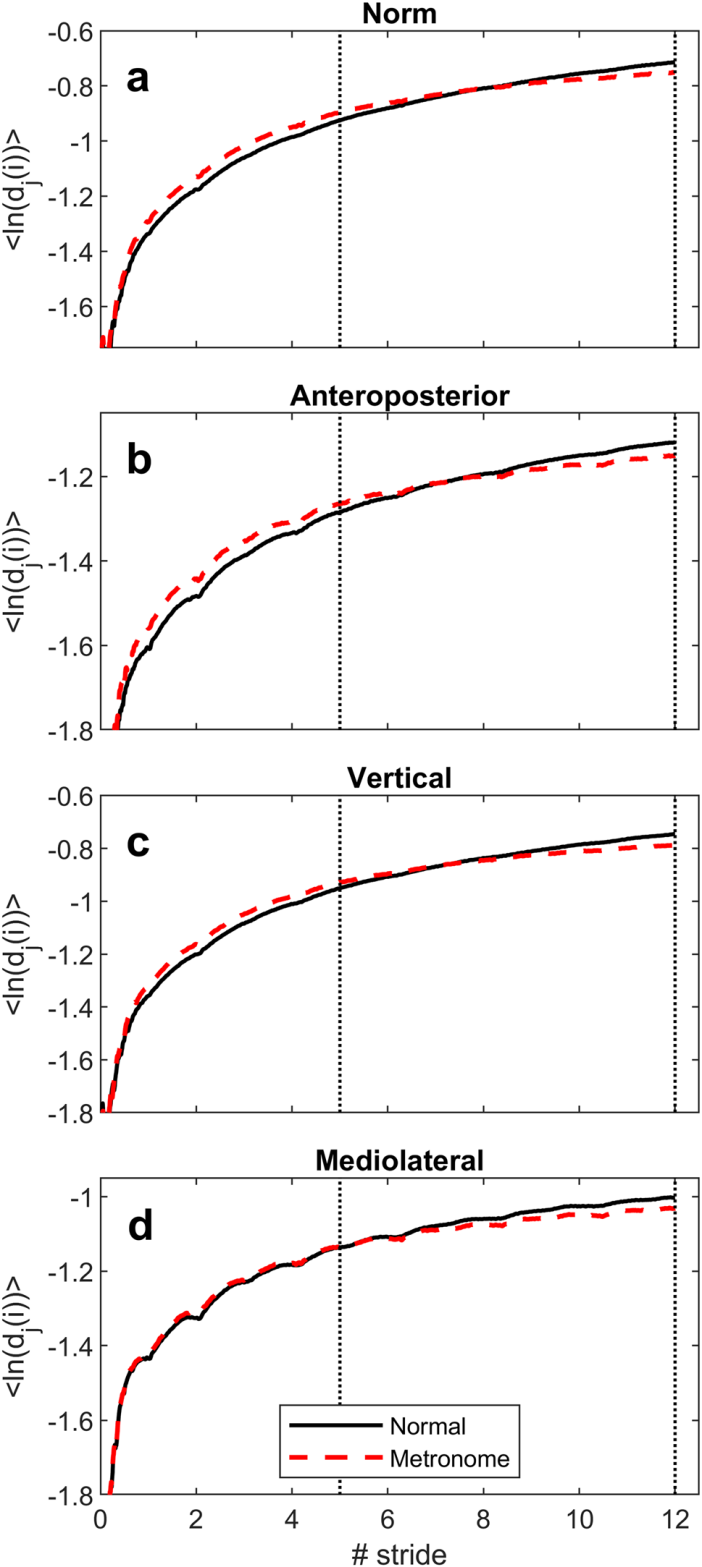
Divergence curves. The figure displays the average logarithmic divergence curves of gait dynamics obtained from the triaxial accelerometer attached to the lower back. The first subplot (**a**) shows the outcome of the vector norm (vector magnitude), while other subplots (**b**–**d**) represent each axis of the accelerometer. The curves are the averaged results of 58 participants for both normal and metronome walking. The figures display time on the x-axis, normalized by stride duration, and logarithmic divergence on the y-axis. Logarithmic divergence represents the natural log of the distance (*d*) between the *i*-th point downstream of the *j*-th pair of nearest neighbors in the state space (or attractor), averaged over all neighbors. The vertical dotted lines show the range over which the long-term divergence (or attractor complexity index) was computed.

The LDS method involves analysis of the phase space (or attractor) of gait dynamics and logarithmic divergence curves, which show the rate at which adjacent trajectories in this space diverge (Fig. 1)^25^. A higher rate of divergence is interpreted as a lower level of gait stability—a lower robustness of gait in response to perturbations—and therefore a higher risk of falling^26^. Short-term divergence, computed between 0 and 1 step, is the better choice for assessing gait stability^38,40^. However, our recent studies^41,42^ clearly demonstrated that long-term divergence (Fig. 1), is influenced by the complexity of inter-stride variability. Although additional investigations are still required to provide a solid theoretical basis for this phenomenon, we hypothesized that the complex fluctuations in gait allow for a greater long-term divergence within the attractor that represents the gait dynamics^25,41^.

Like for other physiological signals, human gait patterns of long duration walking exhibit distinctive, scale-free, fractal-like fluctuations^43–45^ which are indicative of a complex internal organization and adaptive responses to environmental changes^46,47^. Indeed, gait and heart rate^48,49^ variability, brain activity (electroencephalogram, EEG^50^), and blood pressure^49^, exhibit fractal properties. This means that their fluctuations are self-similar across different time scales. In simpler terms, the variance patterns observed at small time intervals are statistically similar to those seen over longer durations. Fractal analysis has emerged as a powerful tool to quantify complex physiological processes, potentially reflecting coordinated interactions between various underlying biological subsystems^49^. Regarding human gait complexity, fractal patterns are characterized by long-range correlations, or statistical persistence, among successive strides. Consequently, strides tend to repeat their lengths, durations, and speeds across multiple cycles^44,51^. Recently, it has been observed that the neuromuscular rhythmic activity of the lower limbs measured during walking also presents a fractal pattern similar to that of gait variability^52,53^. This suggests that the fractal pattern of gait is rooted in neural activity related to motor control.

Detrended fluctuation analysis (DFA) is a common method for evaluating gait complexity and identifying correlation structures in stride intervals or in other gait parameters. It yields a ‘fractal index’, also known as a scaling exponent, which is represented with the Greek letter alpha (α). This index ranges from 0.5 (random correlation structure) to 1 (persistent or fractal correlation structure). Several studies have found an association between a low scaling exponent, aging^54,55^, and fall risk^56–58^. A recent study has demonstrated that older adults with extensive Tai-Chi experience exhibit a higher scaling exponent in comparison to inexperienced controls^59^. This finding is notable because Tai Chi has been shown to be an effective exercise for fall prevention^5,60^. Another study using DFA found that older adults rely predominantly on visual feedback for gait stabilization, which may indicate increased cognitive load^61^. In fact, when confronted with visual disturbances, older individuals exhibit reduced scaling exponents and increased gait instability, in contrast to their younger counterparts. This observation suggests that older adults need to focus more on their gait to counteract destabilization. It is therefore hypothesized that association exists between the degree of voluntary gait control, the degree of caution while walking, and the scaling exponent^56,62^. A theoretical framework could suggest that aging affects balance abilities, resulting in adaptive behaviors such as increased caution. As an example of the relationship between attentional demand and scaling exponents, a recent study assessed the correlation structure of gait in patients with chronic low back pain^63^. The study found that low-back pain patients had lower scaling exponents during walking when compared to matched pain-free controls. However, the scaling exponents increased when the patients were visually distracted. This may suggest that patients must pay more attention to gait control to reduce pain. However, this strategy is disrupted when they need to focus on other tasks.

The association between correlation structure between strides and voluntary gait control is further highlighted when examining the impact of external rhythmic stimuli, such as metronome walking or aligning steps with visual cues. These stimuli tend to decrease the scaling exponent due to the precise control and cognitive load required^51,64^. Indeed, under conditions of metronome walking, stride intervals fluctuate around the imposed pace causing the scaling exponent to drop below 0.5 (statistical anti-persistence or anti-persistent correlation structure)^44,65^. Furthermore, experimental treadmill studies have demonstrated a clear correlation between the level of attention directed towards gait and the anti-persistence observed in stride-to-stride fluctuations^64,66^.

While the use of scaling exponent appears promising for assessing the level of voluntary gait control and early fall-risk detection^55–57,59^, its clinical application is hindered by practical challenges. The DFA method requires long series of stride intervals which may be difficult to collect in older patients^67,68^. In-lab data collection techniques, like instrumented treadmills or 3D video analysis^69^, provide ample data, but their field applicability is not possible. In addition, the need to accurately capture stride lengths or durations is a significant hurdle, especially when using a single lumbar accelerometer. Therefore, alternative methods for assessing the correlation structure of gait need to be developed to overcome the limitations of DFA.

DFA and LDS methods offer insight into distinct aspects of gait control and may complement each other in providing valuable information about walking abilities and gait disorders. Indeed, the correlation structure between strides is related to the degree of attention to gait control^64,66^, while the short-term attractor divergence of gait dynamics is related to gait stability and dynamic balance^40,70^. As applied to gait analysis, these two approaches are based on different perspectives: DFA views gait as a series of discrete gait events, while LDS views gait as a continuous quasi-periodic process that repeats over time. However, the discrete gait time series used to study the correlation structure of gait using DFA is the output of a continuous process in which motor control coordinates muscles and joints over multiple cycles. This continuous process produces stride intervals, lengths, and speeds as outputs. This raises the question of whether it is possible to trace the long-term gait fluctuations in a continuous signal that encapsulates both intra- and inter-stride gait dynamics. It seems that the analysis of long-term divergence obtained with the LDS method can achieve this goal.

In 2013, we proposed that the attractor of gait dynamics (LDS) may contain information about the correlation structure of gait parameters in successive strides (DFA)^25^. We suggested that, when gait is synchronized to a metronome, there is a shift to oscillations around a set target value, and thus the long-term divergence is damped more quickly within a narrowly bounded attractor. We^25,41,42,71^, and others^72,73^, have repeatedly found that scaling exponents and long-term attractor divergence exponents (DE) are correlated and respond similarly to metronome walking. A more recent study further demonstrated the sensitivity of long-term divergence to changes in the correlation structure between strides^41^. The study used a modeling approach that simulated well-defined correlation structures (i.e., constant, random, persistent, and anti-persistent structure), thereby mitigating potential biases related to human factors or experimental design.

Although both short-term and long-term DEs are derived from attractor divergence curves (as shown in Fig. 1), their interpretation should therefore be different. In light of this distinction, a new nomenclature has been proposed. Since the long-term DE measures the signal complexity from a multidimensional attractor, the descriptor "Attractor Complexity Index (ACI)" is proposed. However, a validation study is still needed in the target population (older adults) using a lumbar accelerometer that could be used in unsupervised gait quality assessment.

This article presents preliminary results from the “attractor complexity index empirical rationalization” (ACIER) study. The primary goal of ACIER was to validate the ACI as an indicator of gait automaticity and cautiousness in older adults for the early identification of older individuals at risk for falls. The specific aims were to: (1) contrast the responsiveness to metronome walking of the DFA with that of the ACI; (2) determine the reliability of the ACI; (3) determine if the ACI can effectively characterize the gait associated with aging; and (4) assess the ability of the ACI to discriminate, both retrospectively and prospectively, individuals at higher risk of falling.

Our focus here will be limited to presenting results relevant to the first two objectives outlined above. Additionally, we aimed to provide a more detailed characterization of the parameters required for optimal computation of the ACI algorithm. To achieve this, we conducted an experiment to measure lumbar acceleration in older adults during 4 × 200 m indoor walking using a triaxial accelerometer. The walks included both standard and metronome walking. From the resulting acceleration signals, we calculated both short-term and long-term DEs. For comparative analysis, a secondary accelerometer was used to record foot acceleration. This allowed the detection of strides and the subsequent calculation of scaling exponents using DFA. Our analysis then compared the responsiveness of short-term DE (LDS), long-term DE (ACI), and scaling exponent (DFA) to metronome walking. We examined the correlation between these variables and assessed the reliability of both the DEs and the scaling exponent using a test–retest approach. We also assessed the best range for calculating long-term divergence and determined the axis along which the ACI provided the best contrast. Furthermore, we sought to determine whether the integration of 3D accelerations into vector norm for the computation of ACI could be also an effective means of distinguishing between normal and metronome walking.

## Results

### Participants

Of the 143 potential candidates approached for the study, 60 were ultimately enrolled, meeting the target sample size. The reasons for non-participation were failure to meet the inclusion criteria due to age or the presence of a severe gait disorder. In addition, many individuals declined to participate for personal reasons such as distance from the study site, lack of interest in the research, or time constraints.

The mean age of the participants (N = 60) was 76 years, with a standard deviation (SD) of 6 years, ranging from 65 to 88 years. Twelve participants were between 65 and 70 years of age, and 21 were over 80 years of age. In terms of sex distribution, 60% (n = 36) were female and 40% (n = 24) were male. The mean body mass of the participants was 74 kg (SD = 16), and their mean height was 1.68 m (SD = 0.08).

Only five participants reported significant pain with walking, scoring greater than 10 on the 0–100 visual analog scale (VAS). The highest reported pain intensity for one individual was 55/100 on this scale. The majority of participants found it easy to synchronize their gait with the metronome set to their preferred cadence (i.e., the preferred step rate or step frequency). Only 16 participants rated their difficulty as greater than 10 on a 0–100 VAS. Two individuals reported that they experienced significant difficulty maintaining the prescribed cadence, scoring over 75 on the VAS, while ten participants reported mild difficulty, with VAS scores between 10 and 30.

Our study had minimal missing data. However, technical complications with the lumbar accelerometer caused the loss of three recordings. In addition, two participants were unable to complete the corridor walk due to fatigue, which prevented us from determining their preferred walking speeds. Nevertheless, the length of the recordings was sufficient for calculating gait parameters, at least in one of the two laps. A detailed breakdown of the sample size for each variable can be found in Table 1.

**Table 1:** Descriptive statistics and effect sizes.

	Normal walking			Metronome Walking			Effect size	Confidence intervals	
	N	Mean	SD	N	Mean	SD	g (paired)	CI low	CI high
Basic gait parameters									
Walking speed (m/s)	58	1.27	0.24	58	1.26	0.24	0.02	− 0.06	0.10
Step frequency (Hz, step/s)	59	1.89	0.15	59	1.90	0.15	0.03	− 0.02	0.09
Short-term attractor divergence									
LDS-AP	59	0.99	0.45	58	1.00	0.47	0.02	− 0.26	0.29
LDS-V	59	1.04	0.51	58	1.15	0.55	0.18	− 0.12	0.50
LDS-ML	59	1.29	0.32	58	1.25	0.34	− 0.12	− 0.37	0.15
Long-term attractor divergence									
ACI-N	59	0.028	0.010	58	0.020	0.010	− **0.82**	− **1.22**	− **0.47**
ACI-AP	59	0.022	0.009	58	0.016	0.008	− **0.74**	− **1.17**	− **0.33**
ACI-V	59	0.027	0.010	58	0.020	0.010	− **0.78**	− **1.18**	− **0.42**
ACI-ML	59	0.018	0.009	58	0.015	0.010	− **0.37**	− **0.65**	− **0.12**
Correlation structure (foot sensor)									
Alpha (DFA)	60	0.74	0.17	60	0.39	0.22	− **1.75**	− **2.59**	− **1.09**

Sample sizes (N), means, standard deviations (SD), and standardized effect sizes (Hedges’ g) with 99% confidence intervals (CI) of gait parameters measured from lumbar triaxial accelerometer (local dynamic stability LDS, attractor complexity index ACI) and from foot accelerometer (scaling exponent alpha, detrended fluctuation analysis DFA). N, norm; AP, anteroposterior; V, vertical; ML, mediolateral. Significant results are indicated in bold (*p* < 0.01).

### Divergence curves

Figure 1 shows the average logarithmic divergence curves derived from the reconstructed attractors of the gait dynamics (maximal Lyapunov exponent method, Rosenstein’s algorithm), considering all participants together. It’s obvious that the long-term divergence varies between conditions. The curve for metronome walking appears to increase more gradually than that for normal walking. This suggests a reduced rate of divergence, resulting in a lower long-term DE (a.k.a. ACI).

### Tuning Rosenstein’s algorithm

When using Rosenstein’s algorithm to determine DEs, a critical step is to perform a linear fit to the divergence curve, as shown in Fig. 1. We evaluated various range parameters for the linear fit to optimize the tuning of the algorithm, with detailed results presented in the Supplementary Material (see Supplementary Table 1). Four critical observations emerged from the analysis: First, ACI derived from the mediolateral axis was less responsive to metronome walking compared to other axes and the combination of all axes with vector norm (i.e., vector magnitude). Second, increasing the distance downstream from the initial trajectory separation point (i.e., two points that are nearest neighbors in the reconstructed attractor) for applying the linear fit was found to be associated with greater responsiveness to metronome walking, as indicated by a larger standardized effect size (ES). Third, linear fits performed closer to the initial separation point showed greater reliability, as reflected by higher intraclass correlation coefficients (ICCs). Fourth, longer linear fit intervals were associated with greater reliability (higher ICCs). Synthesizing these results, the 5–12 interval was selected for subsequent analyses. This interval demonstrated optimal performance, particularly for the vector norm, where it yielded the highest average sensitivity (ES = − 0.82) while maintaining robust reliability (ICC = 0.67).

### Responsiveness to metronome walking

**Figure 2:**
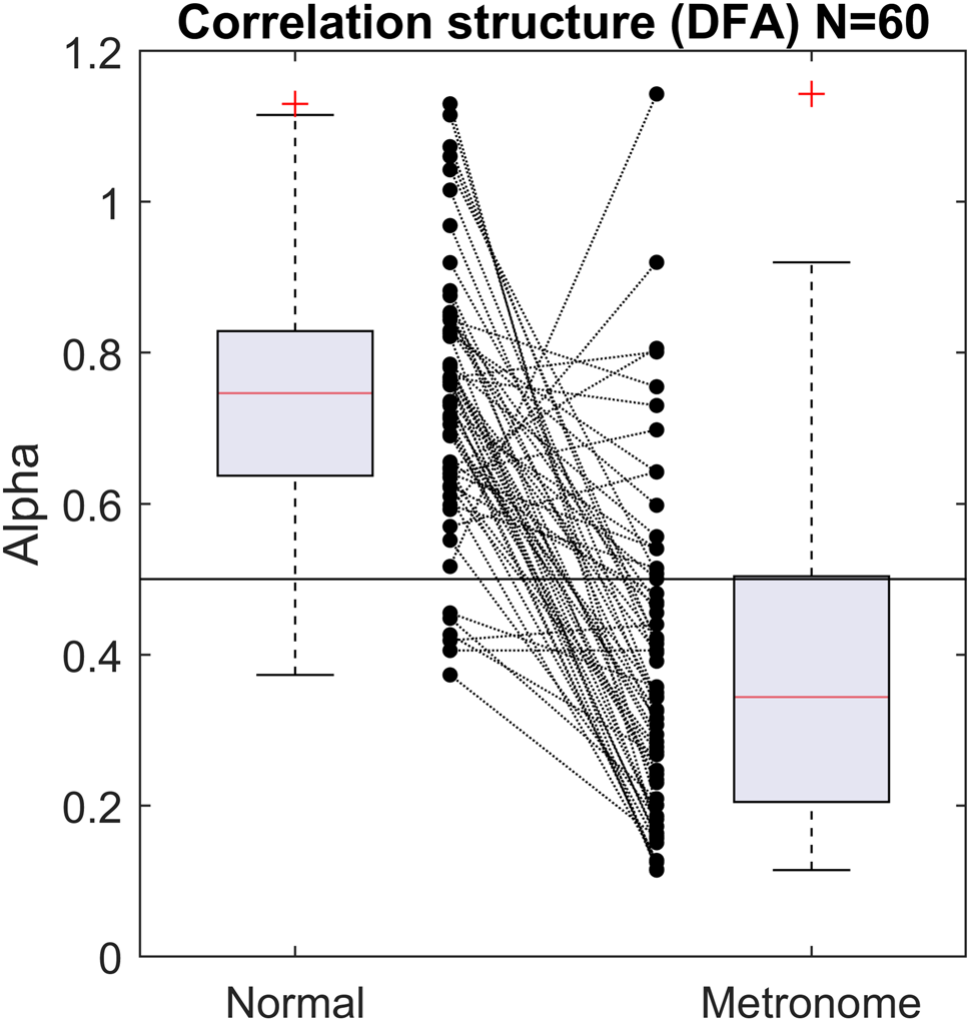
Descriptive statistics of the scaling exponents. The correlation structure (statistical persistence/anti-persistence) among consecutive strides, represented by the scaling exponent alpha, was assessed using the foot accelerometer and detrended fluctuation analysis (DFA). Boxplots show medians, quartiles, range of the data, and outliers ( +) for both normal and metronome walking conditions. Black dots (•) show individual results connected by dotted lines.

**Figure 3:**
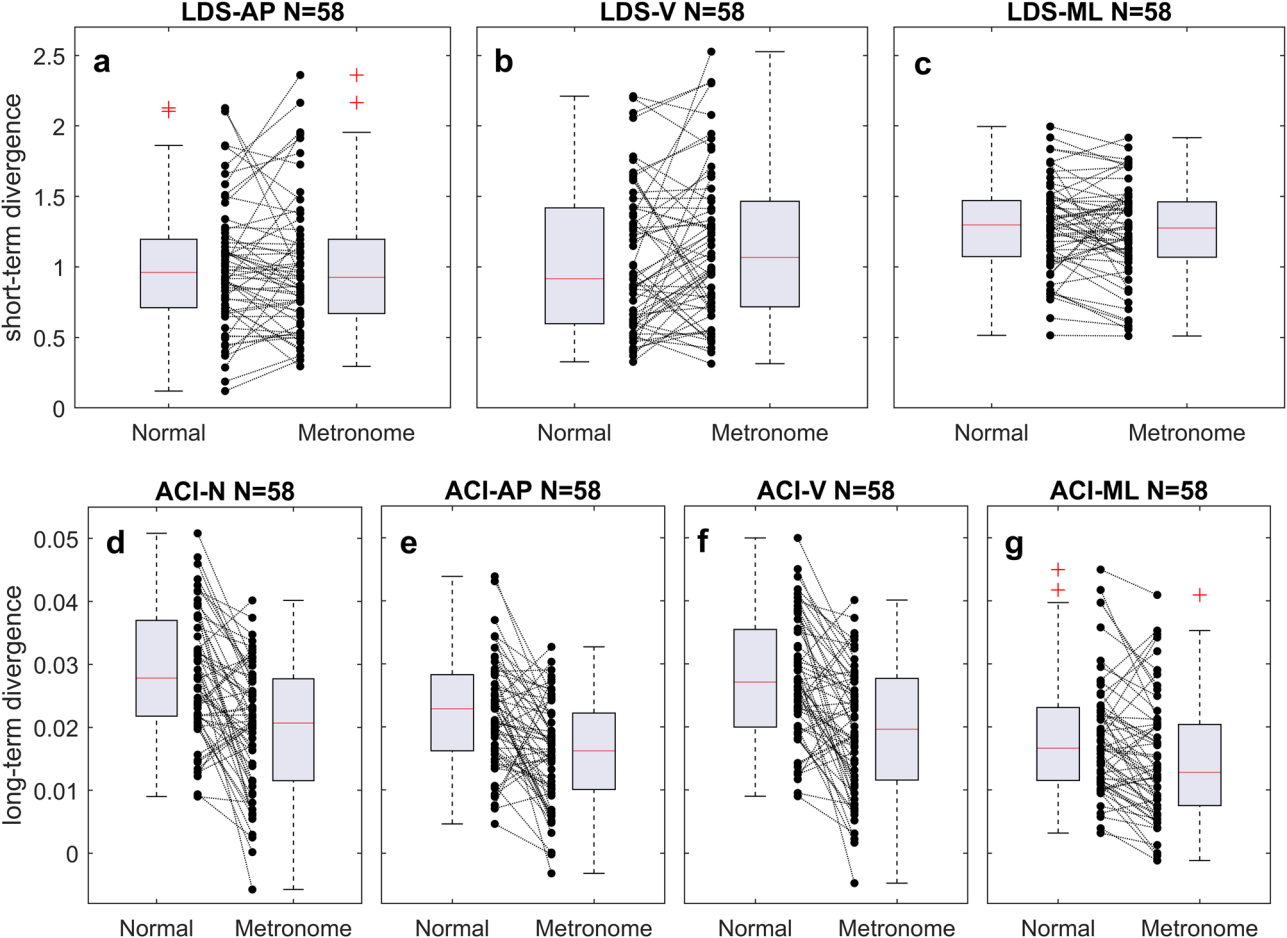
Descriptive statistics of the divergence exponents. The local dynamics stability (LDS, short-term divergence rate), and the attractor complexity index (ACI, long-term divergence rate) were assessed using the lumbar triaxial accelerometer and the Rosenstein’s algorithm for computing the maximal Lyapunov exponent. Subplots show results for vector norm (ACI only, **d**) anteroposterior (AP, **a** and **e**), vertical (V, **b** and **f**) and mediolateral (ML, **c** and **g**) axes. Boxplots show medians, quartiles, range of the data, and outliers ( +) for both normal and metronome walking conditions. Black dots (•) show individual results connected by dotted lines.

Figures 2 and 3 show the distribution of individual results using scatter plots and box plots for DFA (derived from the foot accelerometer) and divergence analysis (derived from the lumbar accelerometer), respectively. In Fig. 2, the DFA results highlight the expected shift from a correlated pattern in normal walking (α > 0.5) to an anticorrelated pattern (α < 0.5). Figure 3 shows that the short-term divergence (LDS, Fig. 2a–c) appears to be unaffected by walking conditions, regardless of the measurement axis in focus. Conversely, the long-term divergence (ACI, Fig. 3d–g) shows a clear difference for vector norm, and in both the antero-posterior and vertical axes.

Synchronizing to the metronome tempo did not significantly change the participants’ walking speed, as shown in Table 1. The average speed difference between conditions was only 0.01 m/s. Similarly, cadence remained consistent across conditions, with a negligible difference of 0.01 step/s. This expected consistency implies that participants also maintained a comparable step length.

In confirmation of descriptive statistics (Fig. 3a–c), when evaluating the short-term DE—an indicator of gait instability and potential fall risk—we observed no significant differences across the three axes (Table 1). The most pronounced standardized effect was quantified at 0.18 on the vertical axis, where short-term DE changed from 1.04 to 1.15. However, the 99% confidence interval (CI) ranging from − 0.12 to 0.50 suggests that the data do not support the rejection of the null hypothesis.

Conversely, long-term DEs, postulated as indicators of gait automaticity and caution while walking, showed a marked decrease when participants synchronized their step frequency to the metronome tempo. The 99% confidence intervals indicate rejection of the null hypothesis that there were no differences between conditions on the three axes and for the norm. The most pronounced effect was for the vector norm, where the DE decreased from 0.028 to 0.020, for a standardized effect size of − 0.82 (Table 1). The effect for the vertical axis was close (0.027–0.020, effect size = − 0.78). The effect for the vertical axis was slightly lower (0.022–0.016, effect size = − 0.74).

As expected, the DFA showed a persistent correlation structure of stride fluctuations during normal walking (with a scaling exponent of α = 0.74). During metronome walking, this shifted to an anticorrelated (or anti-persistent) structure (α = 0.39). The associated standardized effect size was significant at − 1.75.

### Correlations

All bivariate scatterplots and correlation coefficients (Pearson’s r) for gait variables are available in the Supplementary Figure file. Pooling results from both conditions (Fig. S1, N = 117), significant correlations were found between long-term DEs (ACI) and scaling exponents α (DFA) for the vector norm (r = 0.56, *p* < 0.01), and in the anteroposterior (r = 0.46, *p* < 0.01), vertical (r = 0.53, *p* < 0.01), and mediolateral (r = 0.29, *p* < 0.01) axes. No other variables showed significant correlations with the scaling exponent α. In the normal walking condition (N = 57, Fig. S2), correlations between α and ACI were significant at 95% level for the vector norm (r = 0.41, *p* < 0.01), the anteroposterior axis (r = 0.30, *p* = 0.02), and the vertical axis (r = 0.36, *p* < 0.01). For the metronome walking condition (N = 58, Fig. S3), the correlations between ACIs and αs were significant for all the axes and the norm: norm r = 0.46, *p* < 0.01; anteroposterior r = 0.35, *p* < 0.01; vertical r = 0.44, *p* < 0.01, and mediolateral r = 0.32, *p* = 0.016. Upon analyzing the bivariate scatterplots, no indications of spurious correlations due to outliers or nonlinear relationships were observed.

A notable correlation when considering both conditions was the positive relationship between preferred walking speed and the ACI norm (Fig. S1, N = 115, r = 0.30, *p* < 0.01), as well as ACI in the vertical axis (r = 0.32, *p* < 0.01). These correlations were even stronger in the normal walking condition (Fig. S2, N = 57, norm r = 0.52, *p* < 0.01, vertical r = 0.53, *p* < 0.01). ACI measured along the anteroposterior axis also showed a significant, but weaker, correlation in normal walking condition (Fig. S2, N = 57, r = 0.34, *p* = 0.01).

### Reliability

**Figure 4:**
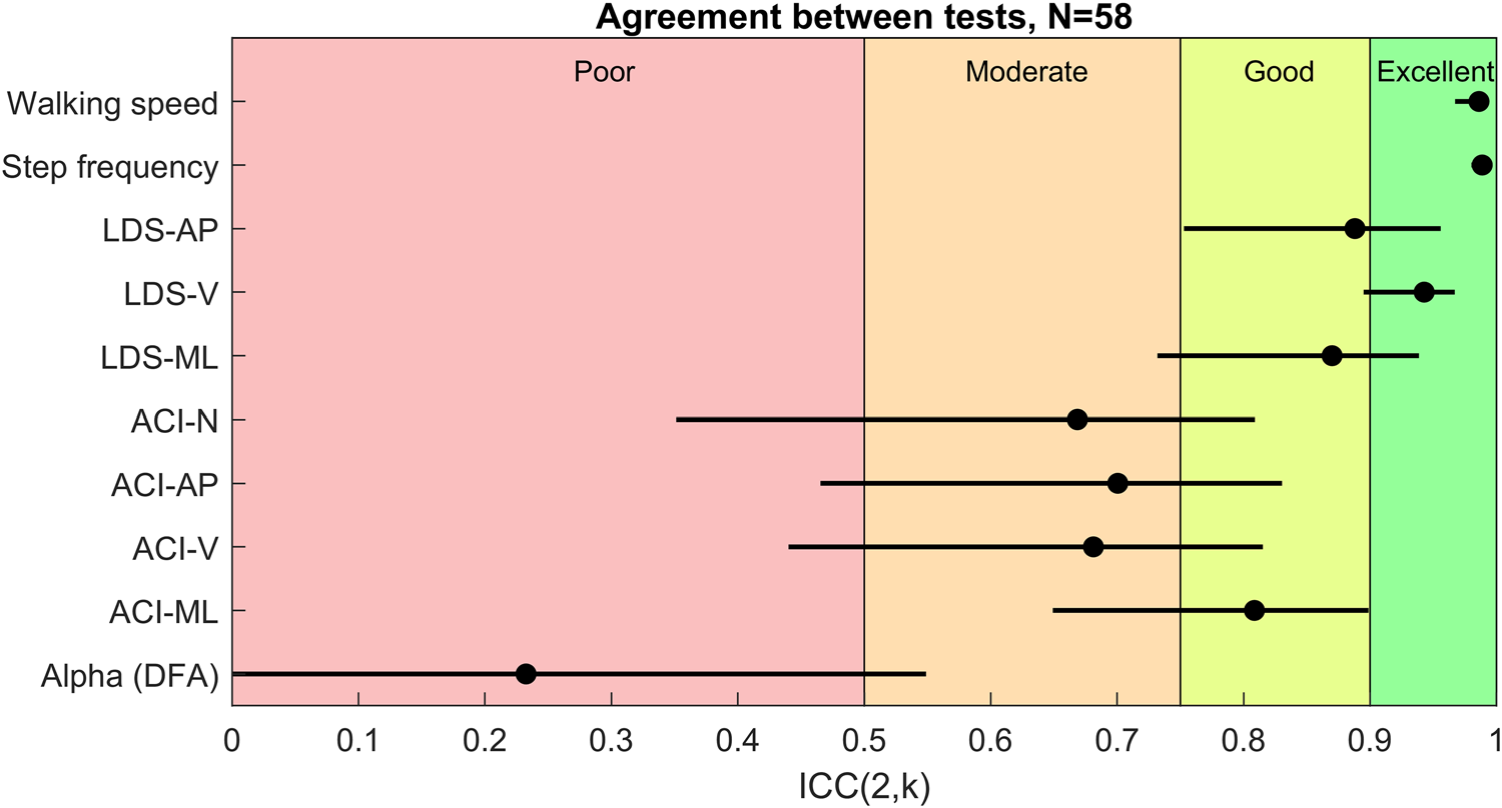
Reliability of the gait parameters. The figure shows intraclass correlation coefficients (ICC) and 95% confidence intervals of gait parameters. LDS: local dynamic stability. ACI: attractor complexity index. N: norm; AP: anteroposterior; V: vertical; ML: mediolateral; DFA: detrended fluctuation analysis.

Table 2 presents the results of the test–retest analysis (reliability study) using intraclass correlation coefficients (ICC), standard errors of measurement (SEM), and smallest detectable difference (SDD). Only the data of the normal walking were analyzed. Figure 4 graphically illustrates the reliability results to enhance interpretation.

**Table 2:** Reliability and absolute consistency.

		Reliability			Absolute consistency	
	N	ICC(3,k)	CI low	CI high	SEM	SDD
Basic gait parameters						
Walking speed	58	0.99	0.97	0.99	0.028	0.078
Step frequency	58	0.99	0.98	0.99	0.017	0.047
Short-term attractor divergence						
LDS-AP	58	0.89	0.74	0.95	0.15	0.40
LDS-V	58	0.94	0.89	0.97	0.12	0.34
LDS-ML	58	0.87	0.71	0.94	0.11	0.32
Long-term attractor divergence						
ACI-N	58	0.67	0.35	0.81	0.0057	0.0158
ACI-AP	58	0.70	0.46	0.83	0.0047	0.0130
ACI-V	58	0.68	0.42	0.81	0.0055	0.0153
ACI-ML	58	0.81	0.65	0.90	0.0041	0.0113
Correlation structure (foot sensor)						
Alpha (DFA)	59	0.23	0.00	0.54	0.15	0.42

Sample size (N), intraclass correlation coefficient (ICC) and 95% confidence intervals (CI), standard error of measurement (SEM), and smallest detectable difference (SDD) of gait parameters measured from lumbar triaxial accelerometer (local dynamic stability LDS, attractor complexity index ACI) and from foot accelerometer (scaling exponent alpha, detrended fluctuation analysis DFA). N, norm; AP, anteroposterior; V, vertical; ML, mediolateral.

In our experimental design, gait parameters such as speed and step frequency showed excellent intra-session test–retest reliability (ICC > 0.95). However, reliability was lower for variability measures. Short-term DEs showed a range of moderate to excellent agreement. Notably, the mediolateral axis, often used to assess dynamic stability and fall risk, had an ICC of 0.87 (95% CI 0.71–0.94). On the other hand, long-term DEs showed moderate to good reliability. Both the anteroposterior and vertical axes had comparable ICC values of 0.70 and 0.68 (CI 0.46–0.83 and CI 0.42–0.81, respectively). ICC for the vector norm was close at 0.67 (CI 0.35–0.81). For the mediolateral axis, the ICC was slightly higher at 0.81 (CI 0.61–0.90). Conversely, the assessment of the gait correlation structure from foot acceleration using DFA showed poor reliability, with an ICC of 0.23 and a wide corresponding CI (0.00–0.54).

In terms of absolute reliability measures (consistency), the basic gait parameters showed excellent agreement between trials. The SDD for speed was 0.078, suggesting that a relative change of 6% (SDD divided by mean) could be interpreted as an actual change. The agreement for other variables was notably lower. For the mediolateral short-term DE, the relative SDD was 25%. For the long-term DEs, the relative SDDs were 59%, 57%, and 62% for the anteroposterior, vertical, and mediolateral axes, respectively. For the vector norm, the relative SDD was 56%. Finally, for the correlation structure determined with the foot accelerometer (scaling exponent α), the SDD was 57%.

## Discussion

In the ACIER study, our focus was to validate the ACI as a measure of gait automaticity and cautiousness using a single accelerometer, particularly in the context of older adults prone to falls. In line with our primary goal, we sought to measure lumbar acceleration during both standard and metronome walking in older adults. Our findings indicate that synchronizing gait to a metronome leads to a discernible decrease in long-term DEs (referred to as ACI), as well as a concomitant decrease in scaling exponents (α). Furthermore, scaling exponents and long-term DEs were correlated. In parallel, short-term DEs seemed not responsive to metronome walking, and were not correlated to scaling exponents. Finally, in a test–retest design, the intrasession reliability of the ACI appeared to be more robust than that of the DFA.

In our analysis of the data processing and algorithmic aspects of the study, it became evident that mediolateral acceleration may not be suitable for evaluating gait automaticity. The Supplementary Table clearly demonstrates that its responsiveness to metronome walking was consistently inferior to that observed in the anteroposterior and vertical axes. This finding can be juxtaposed with the enhanced sensitivity of mediolateral acceleration to dynamic stability documented in prior research^32,38^. This emphasizes that short-term DE and long-term DE reflect separate dimensions of gait motor control. Analyzing short-term divergence in the mediolateral axis offers insight into the gait’s resilience to perturbations that may cause stumbling or falls. In contrast, examining long-term divergence along the other axes can reveal the correlation structure of stride-to-stride variability, indicating the level of control and cautiousness in the gait. Both vertical and horizontal acceleration seemed to be similarly responsive to metronome walking and had close ICC values, whatever the parameter range for linear fitting was considered (see Supplementary table). Furthermore, the correlation analysis (Supplementary Figures) revealed robust correlations between the ACI values measured across these two axes, with coefficients ranging from 0.76 to 0.86. Although this result suggests that both parameters could potentially be used interchangeably, further research is required to ascertain whether one axis is more advantageous in characterizing aging gait.

The results regarding the vector norm indicate that using a composite index based on the total acceleration magnitude could be a suitable method for monitoring gait quality over an extended period. This is particularly important because the orientation of the sensor may not remain constant throughout the day due to subjects involuntarily moving the device, making post-processing reorientation procedures more difficult. The vector norm ACI is even more responsive to metronome walking than when considering the axis separately (ES = 0.82). Additionally, ACI showed a robust correlation with scaling exponents (r = 0.41–0.56, supplementary material). Further research is necessary to confirm these findings in the context of fall risk assessment and aging gait.

While numerous studies, including our own, have examined the effects of rhythmic auditory stimulation on gait patterns in young adults using DFA, our current study represents the first in-depth analysis of its effects in healthy older adults. In our 2005 study, including eight young adults performing 2 × 30 min of outdoor walking, we were the first to observe that under free walking conditions, stride-to-stride fluctuations in speeds, stride lengths, and step times demonstrated long-range, fractal-like correlations^44^. We also observed, using DFA, that metronome walking shifted step-time fluctuation dynamics to an anti-correlated pattern; the scaling exponents shifted from 0.72 in normal walking to 0.21 in metronome walking. This result was further confirmed in treadmill studies by ourselves^51,74^ and others^64,75^. Regarding overground walking studies, Sejdic et al. conducted an experiment in 2012 with a design similar to ours^73^. They tested 15 young individuals in 15-min walking intervals along an indoor circuit using different rhythmic isochronous stimuli. It was observed that the scaling exponent changed from 0.8 in a normal walking condition to 0.4 with auditory stimulation (metronome walking). The results of the present study are consistent with previous studies in younger adults: we found a decrease in the scaling exponent from 0.74 to 0.39 (Table 1). Both younger and older adults appear to respond similarly when synchronizing steps to rhythmic auditory stimulation. However, a detailed comparison between the two age groups is warranted and will be conducted in the next phase of the ACIER study.

It is not a novel^25^, but not yet widely accepted, concept that both long-term divergence (ACI) and scaling exponents may reflect analogous dimensions of gait. The sensitivity of the ACI to rhythmic auditory stimuli and visual cues, similar to the scaling exponent, has been documented in previous treadmill-based research^25,51^. The aforementioned study by Sejdic et al.^73^ using a lumbar triaxial accelerometer found that the ACI—referred to in their publication as the long-term Lyapunov exponent—changed significantly when subjects walked to a metronome compared to their normal walking patterns. Specifically, the ACI decreased from 0.046 to 0.027 (as estimated graphically), yielding a substantial effect size of − 0.63. Meanwhile, the short-term DE remained unchanged. The present results confirm the marked sensitivity of the ACI to metronome walking, as shown in Fig. 3 and Table 1. Notably, the vector norm showed an ES of − 0.82, while the anteroposterior axis showed an ES of − 0.74, and the vertical axis an ES of − 0.78. In parallel, short-term DEs (LDS) showed ESs close to zero. These results reinforce the notion that the ACI—unlike the LDS—may reflect a comparable aspect of gait dynamics similar to that captured by the scaling exponent in the DFA.

Correlation analysis also supports the hypothesis that ACI and scaling exponent are significantly related. Previous treadmill studies^41,42,72^ have suggested this relationship, but our investigation is the first to empirically validate it in a large cohort of older adults engaged in normal (overground) walking. Disentangling the relationships between these variables is a complex task. The Pearson’s correlation coefficient is influenced by the reliability of the measurements in the variables being correlated^76^. In addition, the phenomenon known as the “restricted range problem^77^” occurs when the variability within the values of the correlated variables is limited: with little variability, the correlation coefficient may not necessarily reflect a weak relationship, but rather the homogeneity of the data sample. These two points suggest that the observed correlation coefficients (Supplementary Figures) may indeed be conservative estimates, potentially underestimating the true strength of the relationship between ACI and scaling exponent. Despite these possible methodological challenges, our results revealed significant correlations. For normal walking (Fig. S2), the correlation values were 0.30 and 0.36 for the anteroposterior and vertical axes, respectively, and 0.41 for the vector norm. These correlations were more pronounced in the metronome condition (Fig. S3), with values of 0.35 (anteroposterior axis), 0.44 (vertical axis), and 0.46 (vector norm). When both walking conditions were analyzed together (Fig. S1), the correlations increased further (0.46, 0.53, and 0.56), likely due to the expanded range of data considered, which mitigated the impact of the restricted range effect. Finally, we observed a positive correlation between ACI and preferred walking speed, especially under normal walking conditions (r = 0.28–0.53, Fig. S2). In other words, participants with a naturally slower walking speed also tended to have lower ACIs. While this association warrants further investigation, it is noteworthy that reduced walking speed is indicative of increased caution in walking^78^. This pattern further suggests that ACI could potentially serve as a valid index for assessing gait cautiousness in older individuals.

We hypothesized that ACI might be more reliable (in the sense that it might require fewer steps to obtain usable results) than DFA in assessing correlation structures between successive strides^42^. DFA relies on the analysis of a series of stride intervals, about 150 points in our study, derived from a peak detection algorithm applied to acceleration signals. This approach is prone to measurement errors and gait variations that could affect the final series and, consequently, the DFA results. In contrast, the Rosenstein algorithm used to calculate the ACI directly uses the entire acceleration signal, which contained 18,750 points in our study. This method is likely to be more resilient to isolated errors or drifts within the signal. However, it is important to recognize that the analysis of the correlation structure of gait fundamentally requires the examination of dozens of consecutive strides. This is due to the underlying assumption of scale-free organization in the gait process over an extended timeframe^45^. Recent evidence strongly suggests that DFA requires more than 500 strides to accurately estimate true scaling exponents^54,67^. This is corroborated by our results, which show a notably low ICC for scaling exponent (ICC = 0.23, Table 2 and Fig. 4). Conversely, the ACI showed greater reliability, with ICCs approaching 0.70 (Table 2 and Fig. 4). This is consistent with previous research. For example, in a landmark study by Kang & Dingwell^79^ involving 20 adults on a treadmill, moderate intrasession reliability (ICC ~ 0.6) was observed for long-term DEs, suggesting that walks longer than five minutes are necessary for accurate measurements. In our treadmill study of 95 participants, we found ICCs for long-term DE of 0.54 when considering 70 consecutive strides^29^. Furthermore, we concluded that walking sessions shorter than 35 strides are fundamentally inadequate for accurate estimation of true long-term divergence exponents, even when results from multiple sessions are averaged. In support of this, we observed low ICCs for long-term DEs (0.22–0.34) in a study similar to the present one, but with 40 young participants, shorter walks, and only 40 strides^80^. Taken together, our analysis suggests that although ACI may require a substantial number of consecutive strides, probably at least 200, for reliable results, this number is substantially lower than the 500 strides required for DFA. Therefore, ACI may be an appropriate alternative to DFA in various contexts.

Analysis of the ICC in test–retest experiments provides insight into the relative reliability of a parameter, indicating its robustness (in terms of high statistical power) for group-level inferences. In clinical applications, such as estimating the effect of an intervention on an individual, absolute measures of agreement such as SEM or SDD are more appropriate. Our results show that ACI has a relatively high relative SDD (about 60%), which may make it difficult to distinguish between a true effect and normal variability. This is consistent with our previous study, which also highlighted the difficulties of using DEs for clinical assessment^29^. In the context of ACI for unsupervised applications, the high SDD could be compensated by combining measurements from multiple walking bouts over a week. Our study of 55 patients with chronic lower limb pain^28^ showed an ICC of 0.71 and a relative SDD of 27% when data from 20 one-minute walking bouts over four days were combined. Theoretically, a relative SDD of 16% could be achieved with data from 40 walking bouts over eight days^28^. Additional research is needed to better determine the reliability of the ACI in the context of unsupervised gait quality assessment in older adults.

To refine the interpretation of our findings, it is critical to consider potential biases that could have influenced the results. In particular, attention must be paid to factors that could affect gait variables beyond synchronization with the metronome. The order of presentation of the conditions (normal and metronome) was not randomized, but systematically normal first. Therefore, it cannot be completely excluded that the observed changes in gait patterns were due to fatigue or habituation rather than to the influence of the metronome. However, there was a five-minute break between each condition, which significantly reduced the potential for order-related bias. Pain while walking is also a factor that affects gait in general^81^ and ACI in particular^28^. Therefore, people in pain may not respond as well to the metronome as people who are not in pain. Since we asked participants about their pain level during the test, we can conclude that the effect on the results was marginal. In fact, only five people (8%) reported some pain, but for most of them it was less than 30/100. Finally, we also sought to assess whether participants had difficulty synchronizing their gait to the metronome’s rhythm. While the subjective rating of synchronization performance may not necessarily correlate with the actual level of synchronization, it is noteworthy that most participants reported minimal difficulty in walking to the metronome beat, despite the limited time allotted for practice prior to the test.

Our study presents findings that we believe are robust and broadly applicable to the active older adult population still living at home. The mean age of our participants, 76 years, is elevated and includes a considerable proportion of individuals over 80 years of age, consistent with the demographic most susceptible to fall^82^. The method for recruiting participants required them to proactively contact our research team, which inherently selected individuals with relatively high cognitive function and a vested interest in fall prevention. While this approach may have resulted in a cohort that is healthier and more physically active than the general population of comparable age, it is this segment that is critical for early identification and prevention of fall risk. Our sample had a slightly higher proportion of women, accounting for 60%, compared to the sex ratio in the Swiss elderly population, which is approximately 55%. This difference may be due to psychosocial factors that predispose women to participate more in research.

## Future research and conclusion

In summary, our comprehensive analysis reveals a significant decrease in both long-term attractor divergence (ACI, ES = − 0.82) and scaling exponents (DFA, ES = − 1.75) during metronome walking. Furthermore, both variables were found to be correlated (r = 0.56). This finding validates the utility of ACI in assessing gait automaticity and cautiousness. In comparison to DFA, ACI stands out for its straightforward measurement approach (lumbar accelerometer). ACI also exhibits superior intrasession reliability (ACI ICC = 0.67, DFA ICC = 0.24) and a reduced need for consecutive steps. ACI is therefore a viable and reliable tool for gait quality assessment in unsupervised settings. This is particularly important for older adults, where continuous, unobtrusive monitoring is critical for early identification of individuals at risk of falling. The upcoming phase of the ACIER study will build upon these findings by analyzing the gait patterns of younger adults in parallel. This comparative study will provide insight into age-related differences in gait variability. Furthermore, we intend to assess the potential of ACI for distinguishing between older adults who have recently fallen from those who have not. In a subsequent longitudinal analysis, we will also examine the fall risk prediction capacity of ACI. Looking ahead, ACI has the potential to enable more precise fall risk assessment and better evaluation of intervention strategies aimed at improving gait automaticity, such as our recently proposed gait training program based on interpersonal synchronization^83^.

## Method

### Setting and design

The study was conducted in the Swiss canton of Neuchâtel (French-speaking Switzerland), which had a population of 177,185 in 2023, of which 35,067 people (or 19% of the total) were aged 65 and over. In 2023, Switzerland had a Human Development Index of 0.962, ranking first in the world^84^. This single-center, cross-sectional study was conducted on the campus of the Haute-Ecole Arc Santé in the city of Neuchâtel. The recruitment and measurement phases ran from October 2021 to June 2023.

### Ethical considerations

The research protocol for this study was submitted to the ethics committee ("commission cantonale d’éthique de la recherche sur l’être humain du canton de Vaud", project ID 2021-01937) in October 2021 and received approval in November 2021. The methodology and procedures of the study were conducted in strict accordance with the tenets of the Declaration of Helsinki and in compliance with the Swiss Human Research Act. All participants were thoroughly informed of the objectives and procedures of the study and provided signed informed consent. They also agreed that their data would be anonymized and made publicly available to ensure both their privacy and the transparency of the research**.**

### Enrolment

We aimed to recruit 60 participants, as described in the following sample size assessment. To accomplish this, we worked with various local associations to disseminate advertisements to the target demographic (see the Acknowledgments section for more details).

The inclusion criteria for this study were as follows: individuals aged 65 years or older, capable of walking for 5 min without the assistance of walking aids, and without severe walking disorders (mild gait dysfunction, such as moderate osteoarthritis, was tolerated). The exclusion criteria included osteoarticular, neurological, or other conditions that significantly affect gait, cognitive or psychological disorders that impair the ability to discern, and severe hearing impairment that may hinder the perception of the metronome. Prior to the gait test session, we collected basic participant information, including age, gender, body mass and height.

### Experiment

Participants were equipped with two triaxial accelerometers (Physilog 6S, Gaitup, Switzerland) of compact size (42.2 × 31.6 × 15 mm) and light weight (15 g). These high-precision devices operated at 16-bit resolution with an accuracy of + /− 8 g and a sampling rate of 256 Hz. The first accelerometer was attached to the instep of the right foot, while the second was attached to the lumbar region, specifically at the L4–L5 level. Participants were asked to wear their own comfortable, low-rise shoes. They were instructed to traverse a 205 m, well-lit, corridor four times (two round trips) at their natural walking pace. Each lap was timed to assess their preferred walking speed. After the first round trip, a five-minute break was provided during which walking cadence was derived from lumbar accelerometer data. For the subsequent round trip, participants synchronized their gait to an electronic metronome, the rhythm of which was set to the cadence determined during the break. Prior to the metronome test, participants performed a short 30-s walk to practice synchronization.

Finally, participants were asked to answer two questions about their pain while walking and the ease with which they could synchronize their gait with the metronome. We used VAS to quantify their subjective pain and feeling, resulting in a score ranging from 0 to 100. A score of zero indicates no pain and no difficulty in following the metronome, while a score of 100 represents the worst pain imaginable and a complete perceived inability to maintain a correct pace.

### Data analysis

Acceleration data were uploaded from the devices to the institutional server. Most calculations and analyses were performed using MATLAB R2021a (The MathWorks Inc., Natick, MA). Some ancillary analyses (reliability) were performed using R (R Core Team, 2020^85^).

By visual inspection of the acceleration signals, segments indicative of steady walking were selected for subsequent analysis. The mean step frequency was derived from the vertical acceleration signal by evaluating its spectrum, which was obtained using a fast Fourier transform.

The stride durations were derived from the foot acceleration signals. First, the norm of the 3D accelerations was calculated. These data were then integrated and detrended to produce a smoother signal indicative of speed. Subsequent application of a peak detection algorithm identified local maxima in the signal, each representing the maximum speed of the foot during a gait cycle. The time between two consecutive peaks was considered as the stride time. DFA was then used to determine the scaling exponent characterizing the stride time series^71^. Box sizes ranging from 16 to N/2 (N = total number of strides) were selected using the evenly spaced algorithm^86^. Our selection of box sizes was influenced by the recent guidelines outlined in the systematic review by Ravi et al.^54^, which were based on the experimental findings of Damouras et al.^68^. However, due to the brief duration of gait tests in our study, we adjusted the upper limit of box sizes to N/2 instead of N/9.

To correct for potential sensor misalignment in the subsequent analysis of the lumbar acceleration, we applied the Moe-Nilssen’s method^87^, which uses the principle that the Earth’s gravitational acceleration should only be detected on the vertical axis of a properly aligned 3D accelerometer. Through trigonometric computations, the method virtually reorients the sensor, ensuring accurate representation of acceleration data by compensating for any deviations from the ideal alignment.

As an alternative to correcting individual acceleration components for misalignments, we calculated the vector norm (also known as vector magnitude) from the 3D acceleration signals. The norm of a 3D vector represents its overall length or strength. It is calculated by taking the square root of the sum of the squares of its individual components. In simpler terms, it captures the vector’s size without considering its direction. This provides a precise measure of acceleration intensity without the need to consider the sensor’s tilt or rotation. This feature is particularly advantageous in situations where there is a possibility that the sensor may shift during the measurement, as in long-duration unsupervised gait analysis.

To assess the short-term and long-term DEs, we analyzed the acceleration signal using the maximal Lyapunov method, implemented through Rosenstein’s algorithm^25,88^. First, signal lengths were reduced to uniformly include 250 steps, using the step frequency to find the correct duration and hence the correct number of samples to keep. Then, the resulting signals were resampled to a constant number of 18,750 samples (75 samples per step) using a polyphase interpolation structure (Matlab built-in function *resample*). This step is important because the Rosenstein algorithm is sensitive to sample length^29,89^. The reconstruction of the attractor of gait dynamics was based on the principles of Takens’ theorem^25,90^. For our analysis, the dimensionality of the state space (embedding dimension) was determined using the global false nearest neighbors (GFNN) method^25,91^, resulting in a five-dimensional state space, which was uniformly applied to all signals. Then, we calculated the time delay for each individual signal using the average mutual information (AMI) method^25,92^. The AMI values were in the range of 15 to 18 samples. Following the reconstruction of the attractor, Rosenstein’s algorithm was then employed to quantify the divergence of nearby trajectories within this state space^25^. We used a custom version of the algorithm implemented to Matlab, which is similar to a script available online^93^. To avoid autocorrelation effects, neighbors close in time are excluded from the nearest neighbor search (Theiler window). The size of this exclusion window was set to 75 samples, which is a step. The time was normalized to the duration of a stride (one stride is represented with 150 samples). The maximum time over which the divergence of neighboring trajectories in the state space was computed was set to 1800 samples (12 strides). Using the resulting logarithmic divergence curves and linear fits (Fig. 1), several time frames were used to calculate the rate of divergence (divergence exponent). In terms of short-term divergence (LDS), we used a 0–0.5 stride range, which has been found to be a good choice for assessing dynamic balance^38^. In terms of long-term divergence (ACI), we explored different combinations of lengths and ranges to better define the optimal parametrization of the algorithm. The full analysis is presented in a supplementary file (Supplementary Table). In the main article, we only presented the best length/range combination.

The variables—preferred walking speed, step frequency, scaling exponent α, short-term DE (LDS), and long-term DEs (ACI) in the three axes and for the norm of the 3D signal—were computed individually for each outward and return lap. For the primary analysis, the values from both laps were averaged. If one of the two laps was missing, the value of the remaining lap was used. However, for the reliability (test–retest) analysis, results from both the outward and return laps were included but not averaged.

### Statistics

In our descriptive statistics, both individual data points and boxplots—highlighting the median, quartiles, and data range—are presented in Figs. 2, 3. Means and standard deviations are shown in Table 1. Differences between conditions were assessed using the standardized effect size, with the goal of facilitating comparisons between variables that have different metrics. We used Hedges’ g methodology^94^, which provides a correction for the biases inherent in smaller samples. To account for correlations between conditions within individuals, we treated results as paired (or dependent). The 99% confidence intervals (CIs) were derived using bootstrapping techniques. It is pertinent to mention that inferential analysis can be performed using CIs: similar to a paired t-test, if the CI does not encompass zero, one can reject the null hypothesis of no difference between conditions.

The decision to use 99% CIs resulted from careful consideration of the correlated nature of our multiple variables. For example, we expect alpha to correlate with ACI, and speed to correlate with stride frequency. Traditional family-wise error rate control methods often assume test independence, making them overly conservative when applied to correlated tests. This conservatism can increase the risk of Type II errors. By opting for 99% CIs, we aimed to strike a reasonable balance: ensuring control of Type I errors associated with multiple comparisons, while acknowledging the inherent correlations between our tests.

In the supplementary material (Supplementary Figures), we performed a systematic analysis of the correlations between gait variables using a linear fit approach based on the least squares method. This analysis was divided into three distinct sub-analyses: the full sample (including both conditions), the normal walking condition, and the metronome walking condition. Bivariate scatterplots and histograms provide a comprehensive visualization of the data distribution. Each plot shows the Pearson’s r correlation coefficient. Results considered significant, where r is statistically different from zero at *p* < 0.05, are highlighted in red.

We used the repetitions of the corridor walks (out and back laps) to assess how reliable the gait variables were in normal walking at least in the short term (intra-session reliability). For the purposes of out-of-lab gait measurements and extended unsupervised evaluations, we did not analyze the results of the metronome walks. This walking modality is typically limited to short-term experiments, and understanding the reliability of gait variables in this specific context is of limited interest.

In a test–retest context, reliability refers to the extent to which measurements yield consistent results over repeated trials. The intraclass correlation coefficient (ICC) is a reliability coefficient that quantifies the proportion of total variance due to between-subjects variability^95^. We sought to determine the absolute agreement between two repetitions, taking into account the mean (as used in the primary analysis)^96^. This approach used a 2-way mixed-effects model, commonly referred to as a 3,k model in Shrout & Fleiss terminology and A,k in McGraw & Wong nomenclature^95^. For our computations, we used the R package “Psych”^97^, which favors mixed models over ANOVA and thus provides improved handling of missing values and unbalanced designs because it relies on maximum likelihood estimations instead of sum of squares. We calculated 95% confidence intervals around the ICC estimates using a bootstrap procedure. To enhance interpretation (see Fig. 4), we adopted the thresholds suggested by Koo & Li^96^: “[ICC] values less than 0.5 are indicative of poor reliability, values between 0.5 and 0.75 indicate moderate reliability, values between 0.75 and 0.9 indicate good reliability, and values greater than 0.90 indicate excellent reliability”.

To complement the ICC analysis, we also computed SEMs. SEM^98^ is a measure of the standard deviation of errors of measurement. As an absolute value of reliability, it quantifies the expected trial-to-trial noise in the data^95^. SEM is essential for deriving clinically meaningful estimates, such as the minimum detectable change or the CIs with true scores^95^. Here (Table 2) we highlight the SDD to provide a better understanding of the reliability of gait measurement in a clinical setting. The SDD represents the smallest genuine change that can be detected, considering the variability of the measure across several trials^95,99^.

### Sample size

Previous treadmill studies have reported standardized effect sizes greater than 2.0 in younger individuals when comparing metronome walking to standard walking conditions using long-term DE (ACI) as a measure^25,42^. Based on this effect size, with an alpha level of α = 0.01 and a power of 1−β = 0.9, the expected minimum sample size was determined to be 8 subjects. However, given the lack of studies evaluating metronome walking in elderly populations, this modest sample size may prove inadequate. Therefore, a target cohort of 60 participants was established in accordance with other objectives of the ACIER study (pending publication). A subsequent sensitivity analysis revealed an expected detectable minimum effect size of 0.51 (with N = 60, α = 0.01 and 1−β = 0.9). As a result, the sample size appeared adequate to meet the primary objective of the study.

### Supplementary Information

Supplementary Figures.

Supplementary Information.

## Data Availability

Raw acceleration data are available on Zenodo: 10.5281/zenodo.10148825^100^.
