# Supplementary material for: Effects of metronome walking on long-term attractor divergence and correlation structure of gait: a validation study in older people
Source: Sci Rep. 2024 Jul 9;14:15784. doi: 10.1038/s41598-024-65662-5 (PMC11233570; doi:10.1038/s41598-024-65662-5)
Supplement: Supplementary file 1 — Supplementary Figures. [file 41598_2024_65662_MOESM1_ESM.pdf]

Effects of metronome walking on long-term attractor divergence and correlation structure of gait: a validation study in older people

Sophia Piergiovanni[1] and Philippe Terrier[1*]

Supplementary figures

Figure S1. Histograms, scatterplots and Pearson's correlation coefficients, all data. Page 2

Figure S2. Histograms, scatterplots and Pearson's correlation coefficients, only normal walking data. Page 3

Figure S3. Histograms, scatterplots and Pearson's correlation coefficients, only metronome walking data. Page 4

© Piergiovanni & Terrier 2024

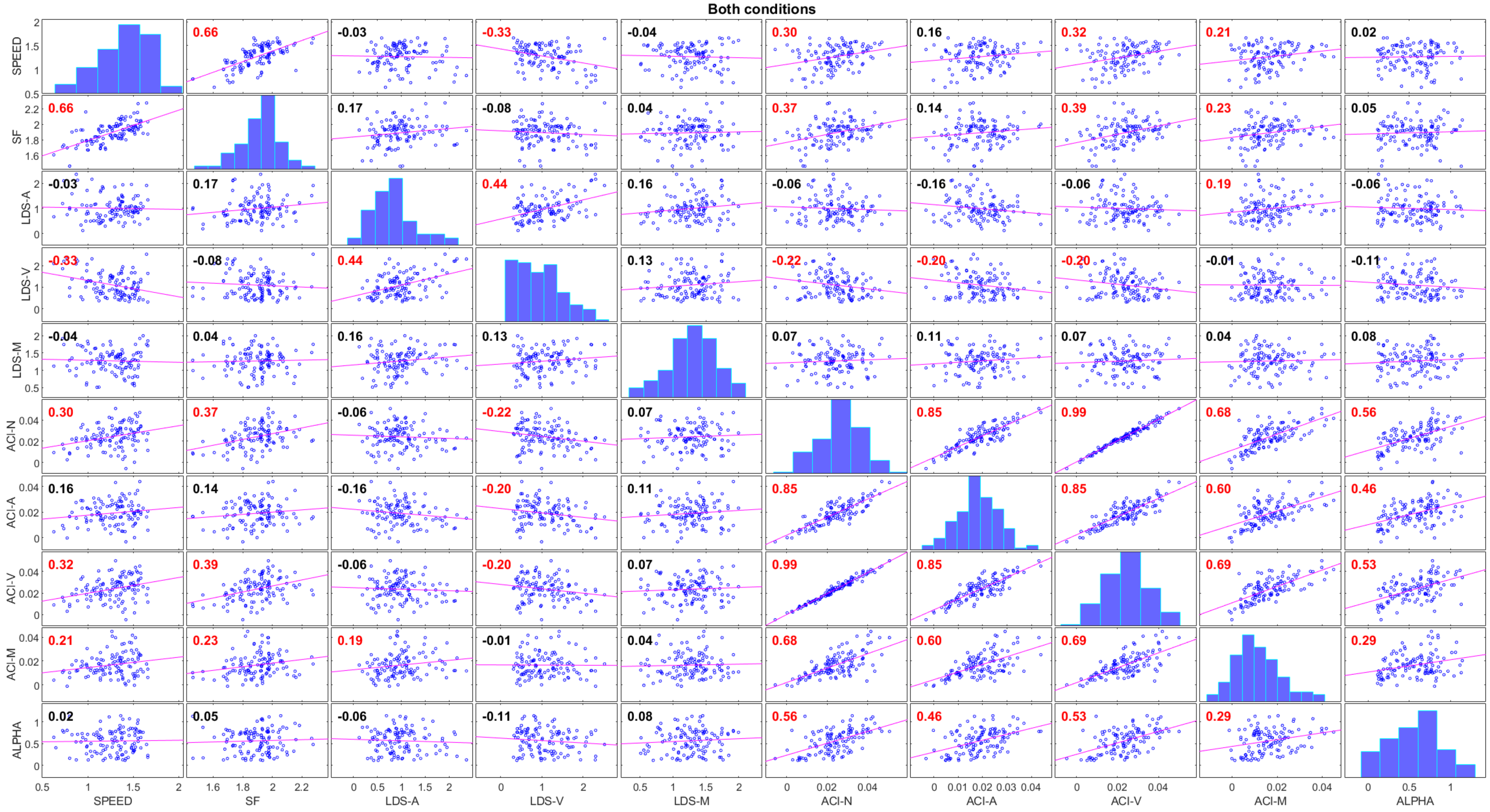

Figure S1. Histograms, scatter plots, and Pearson's correlation coefficient of gait variables for both conditions together (N=117). SF: step frequency. LDS: local dynamic stability (short-term divergence). ACI: attractor complexity index (long-term divergence). Alpha: scaling exponent (detrended fluctuation analysis). N: norm; AP: anteroposterior. V: vertical. ML: mediolateral. Significant correlations (p<0.05) are highlighted in red.

CC BY

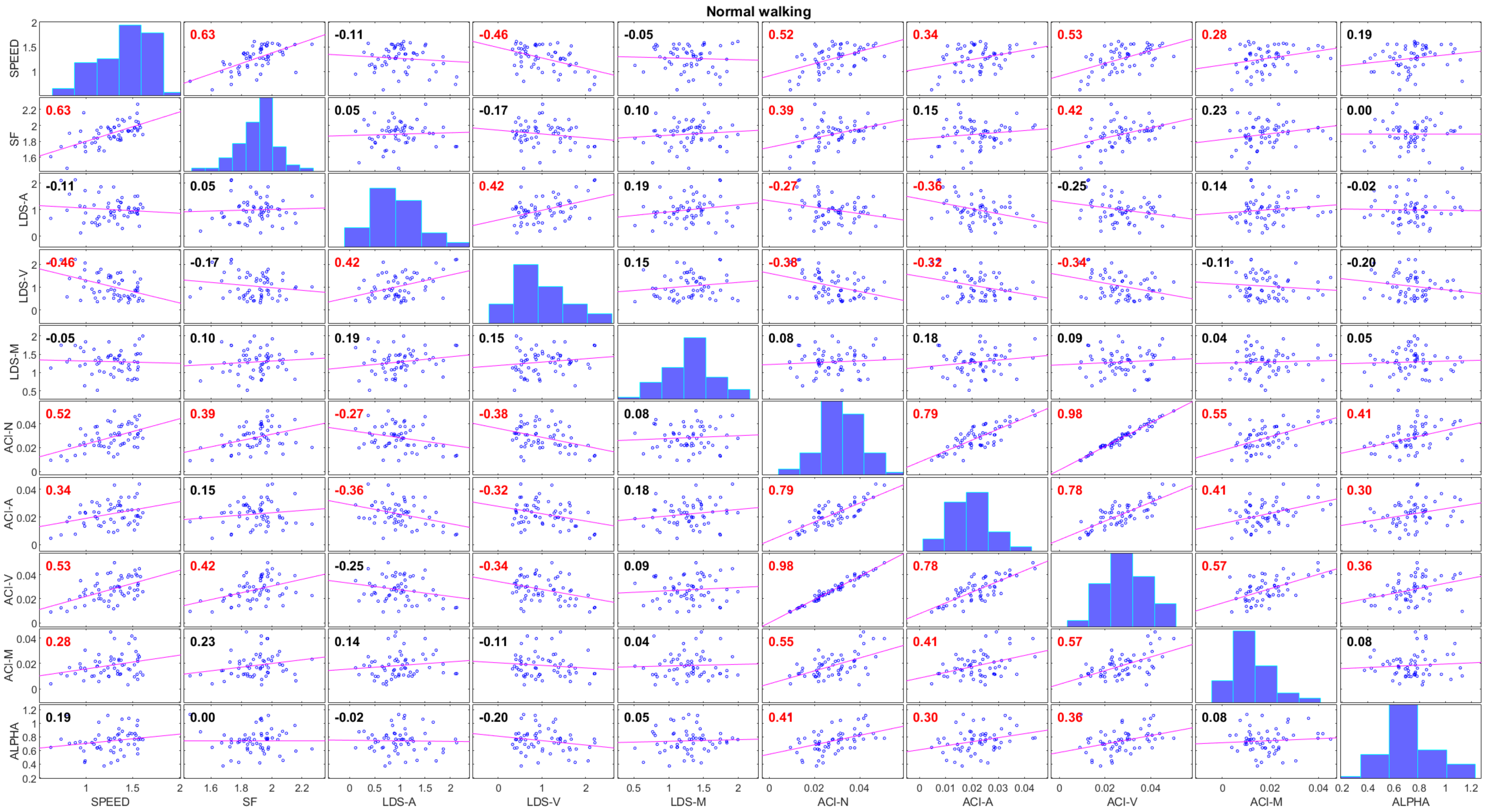

Figure S2. Histograms, scatter plots, and Pearson's correlation coefficient of gait variables for the normal walking condition (N=58). SF: step frequency. LDS: local dynamic stability (short-term divergence). ACI: attractor complexity index (long-term divergence). Alpha: scaling exponent (detrended fluctuation analysis). N: norm; AP: anteroposterior. V: vertical. ML: mediolateral. Significant correlations ($p<0.05$) are highlighted in red.

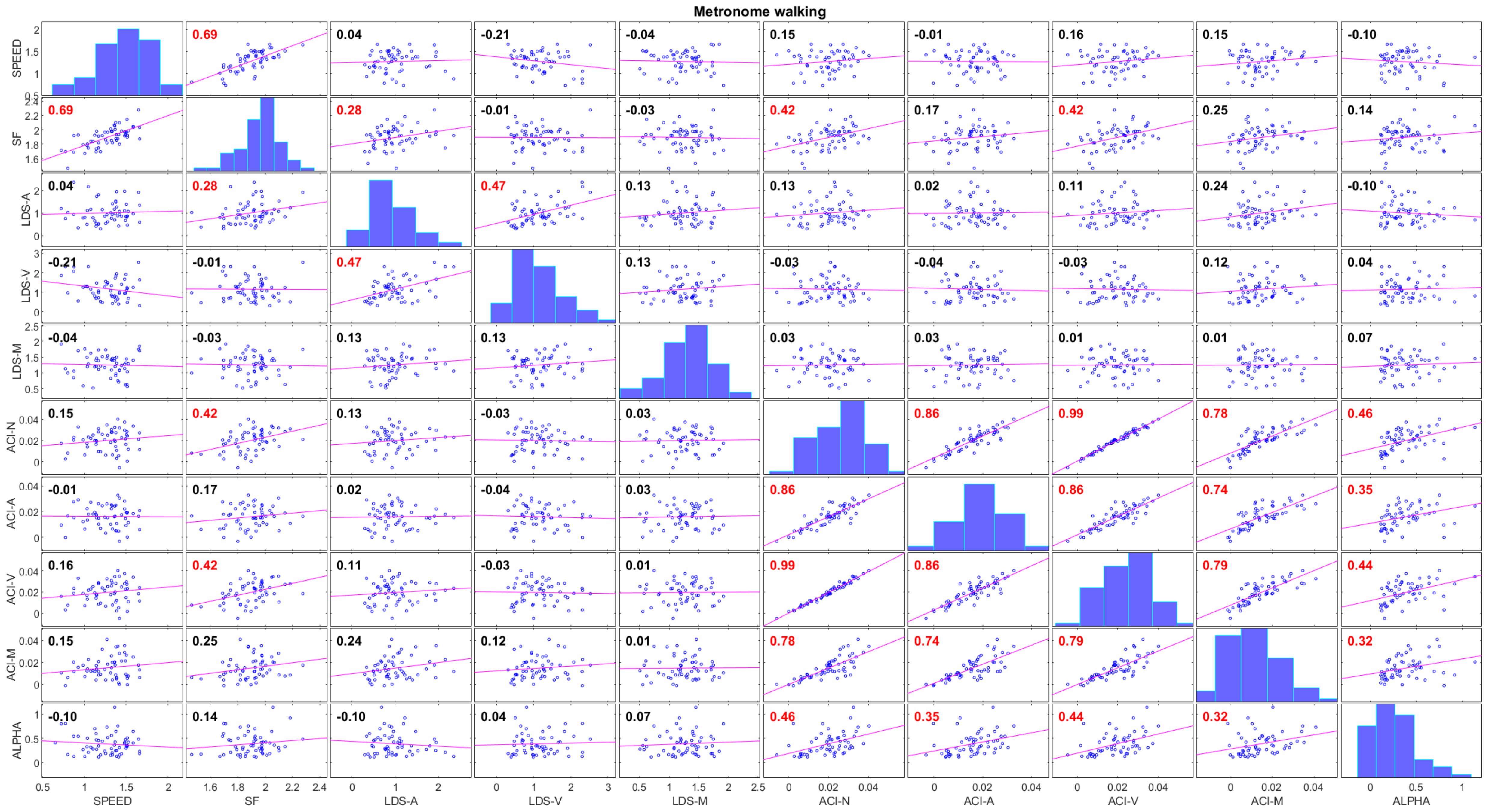

Figure S3. Histograms, scatter plots, and Pearson's correlation coefficient of gait variables for the metronome walking condition (N=58). SF: step frequency. LDS: local dynamic stability (short-term divergence). ACI: attractor complexity index (long-term divergence). Alpha: scaling exponent (detrended fluctuation analysis). N: norm; AP: anteroposterior. V: vertical. ML: mediolateral. Significant correlations (p<0.05) are highlighted in red.

© Piergiovanni & Terrier 2024
