# Supplementary material for: Effects of metronome walking on long-term attractor divergence and correlation structure of gait: a validation study in older people
Source: Sci Rep. 2024 Jul 9;14:15784. doi: 10.1038/s41598-024-65662-5 (PMC11233570; doi:10.1038/s41598-024-65662-5)
Supplement: Supplementary file 2 — Supplementary Information. [file 41598_2024_65662_MOESM2_ESM.pdf]

Effects of metronome walking on long-term attractor divergence and correlation structure of gait: a validation study in older people

Sophia Piergiovanni[1] and Philippe Terrier[1]

[1] Haute-Ecole Arc Santé, HES-SO University of Applied Sciences and Arts Western Switzerland,

Supplementary table

The supplementary table (next page) shows the result of the tuning of the Rosenstein's algorithm to optimally assess the long-term divergence of gait dynamics (a.k.a. attractor complexity index, ACI).

The purpose was to find the optimal range for computing the slope (i.e., the divergence rate) from the divergence curve using linear fitting, as illustrated below. Two outcomes were used: 1) the standardized effect size (Hedges' g) of the difference between metronome and normal walking, defined as responsiveness, presented with 99% confidence intervals; and 2) the intraclass correlation coefficients (ICC), defined as test-retest reliability, presented with 95% confidence intervals. Note that the reliability is computed for normal walking only. See the method section of the main article for further information.

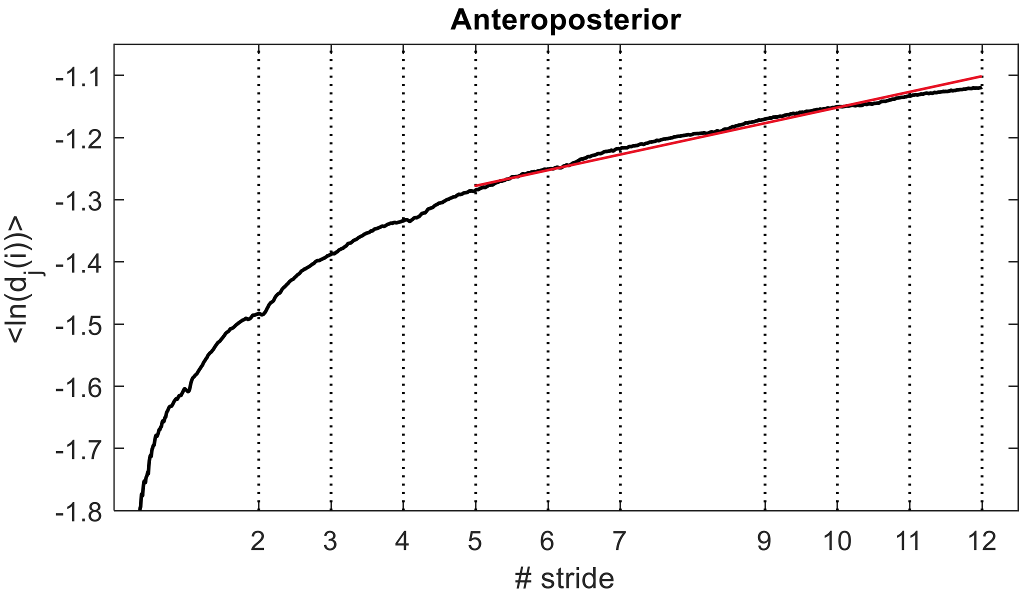

Illustration of a divergence curve with associated linear fit. *This figure shows an example divergence curve with a linear fit, shown in red, to calculate the divergence rate, also known as the divergence exponent. The vertical dotted lines indicate the boundaries used to calculate the various divergence rates, which are detailed in the table below.*

CC BY

			Responsiveness (metronome vs. normal)			Reliability (test-retest)		
			**Effect size**	**CI low**	**CI high**	**ICC**	**CI low**	**CI high**
Length **7**	Range 5–12	ACI-N	-0.82	-1.24	-0.45	0.67	0.36	0.81
		ACI-AP	-0.74	-1.15	-0.36	0.70	0.48	0.84
		ACI-V	-0.78	-1.17	-0.42	0.68	0.44	0.82
		ACI-ML	-0.37	-0.66	-0.13	0.81	0.65	0.90
	Range 4–11	ACI-N	-0.77	-1.16	-0.43	0.71	0.51	0.83
		ACI-AP	-0.71	-1.10	-0.33	0.76	0.59	0.87
		ACI-V	-0.73	-1.15	-0.39	0.73	0.52	0.84
		ACI-ML	-0.37	-0.62	-0.14	0.83	0.71	0.90
	Range 3–10	ACI-N	-0.67	-1.03	-0.32	0.77	0.64	0.86
		ACI-AP	-0.66	-1.07	-0.30	0.78	0.64	0.87
		ACI-V	-0.64	-1.02	-0.31	0.79	0.66	0.87
		ACI-ML	-0.33	-0.56	-0.13	0.84	0.71	0.91
	Range 2–9	ACI-N	-0.52	-0.85	-0.20	0.79	0.66	0.86
		ACI-AP	-0.58	-0.95	-0.23	0.77	0.61	0.86
		ACI-V	-0.49	-0.84	-0.20	0.81	0.71	0.88
		ACI-ML	-0.26	-0.47	-0.06	0.86	0.74	0.92
Length **6**	Range 6–12	ACI-N	-0.82	-1.23	-0.46	0.64	0.26	0.80
		ACI-AP	-0.72	-1.13	-0.33	0.65	0.32	0.79
		ACI-V	-0.78	-1.22	-0.42	0.65	0.35	0.79
		ACI-ML	-0.34	-0.62	-0.06	0.79	0.53	0.88
	Range 5–11	ACI-N	-0.78	-1.16	-0.42	0.69	0.45	0.83
		ACI-AP	-0.72	-1.14	-0.34	0.73	0.53	0.84
		ACI-V	-0.74	-1.16	-0.40	0.70	0.44	0.83
		ACI-ML	-0.37	-0.65	-0.15	0.82	0.65	0.90
	Range 4–10	ACI-N	-0.73	-1.12	-0.39	0.74	0.55	0.84
		ACI-AP	-0.69	-1.11	-0.33	0.77	0.64	0.88
		ACI-V	-0.70	-1.09	-0.36	0.75	0.59	0.84
		ACI-ML	-0.39	-0.66	-0.17	0.82	0.69	0.90
	Range 3–9	ACI-N	-0.62	-0.98	-0.30	0.77	0.66	0.86
		ACI-AP	-0.63	-1.02	-0.27	0.78	0.65	0.87
		ACI-V	-0.59	-0.96	-0.28	0.80	0.69	0.87
		ACI-ML	-0.31	-0.55	-0.11	0.85	0.71	0.91
Length **5**	Range 7–12	ACI-N	-0.85	-1.26	-0.46	0.56	0.12	0.76
		ACI-AP	-0.72	-1.19	-0.31	0.58	0.31	0.76
		ACI-V	-0.79	-1.25	-0.42	0.59	0.22	0.75
		ACI-ML	-0.28	-0.59	0.01	0.76	0.54	0.89
	Range 6–11	ACI-N	-0.78	-1.17	-0.14	0.66	0.28	0.81
		ACI-AP	-0.70	-1.11	-0.30	0.69	0.49	0.83
		ACI-V	-0.74	-1.16	-0.36	0.67	0.35	0.82
		ACI-ML	-0.34	-0.61	-0.10	0.80	0.61	0.89
	Range 5–10	ACI-N	-0.74	-1.12	-0.37	0.71	0.50	0.84
		ACI-AP	-0.70	-1.09	-0.32	0.75	0.58	0.86
		ACI-V	-0.71	-1.09	-0.34	0.71	0.51	0.83
		ACI-ML	-0.41	-0.67	-0.18	0.80	0.65	0.88
	Range 4–9	ACI-N	-0.70	-1.07	-0.35	0.74	0.59	0.83
		ACI-AP	-0.67	-1.06	-0.31	0.78	0.63	0.87
		ACI-V	-0.67	-1.05	-0.34	0.76	0.61	0.85
		ACI-ML	-0.38	-0.65	-0.15	0.83	0.71	0.90

Table S1. Responsiveness and reliability of the ACI algorithm for different ranges and lengths applied to the linear fit of the divergence curves. N=norm, AP=anteroposterior, V=vertical, ML=mediolateral.

© Piergiovanni & Terrier 2024
